# Missing Links in Joint Disease

**Published:** 1903-06

**Authors:** Preston King

**Affiliations:** Honorary Physician, Royal Mineral Water Hospital, Bath.


### MISSING LINKS IN JOINT DISEASE.

BY

PRESTON KING, B.A., M.D., B.C. Camb.,
*Honorary Physician, Royal Mineral Water Hospital, Bath.*

---

MY object in this paper is, in the first place, to dispose of rheumatoid arthritis as a distinct disease, and then to suggest that many of the cases that are at present included under this name are in reality manifestations of the rheumatic state, whilst the rest are the outcome of various infective processes. I think the time has come when it is at least reasonable to doubt whether, as a distinct and seperate disease, rheumatoid arthritis exists at all. For my part, I have come to regard it as a condition which results from various causes.

No one, I feel sure, who attended the section in medicine at the Cheltenham meeting two years ago, when this subject was under discussion, could have failed to be struck by the remarkable divergence of opinion which existed as to what rheumatoid arthritis really was. No two speakers were mutually agreed as to a definition of the disease, or as to what cases it should include. If we turn to the literature upon the subject, the confusion is the same, with this addition, that there are almost as many names under which it is described as there are authors to describe it.

With all these various opinions, and with all this difficulty in classification, it is certainly allowable to ask whether rheumatoid arthritis should any longer be included in the list of separate diseases. It is said to occur at any period of life, from childhood to extreme old age. The typical cases belong to early adult life, and are seen seven times more often in women than in men. With the puffy synovial swellings, especially of the smaller joints; the anxious look, the quickened pulse, the wasted muscles, the increased reflexes, and general debility, we have shortly the clinical picture of a typical case of rheumatoid arthritis. But in the descriptions of this disease there are also included cases which present only those bony changes in the phalanges, which are known as Heberden's nodes; those in which one joint, perhaps the hip, of some old man is semi-ankylosed and fixed; and others where osteo-arthritic changes have affected the vertebræ till the spine has grown together into a deformed and solid mass.

Such, then, are the cases that are at present included under the title rheumatoid arthritis; and, I ask, is it a disease by itself? or are not these symptoms rather the outcome of various causes?

First, then, is it a distinct disease? distinct, that is, as we, rightly or wrongly, apply the term to tuberculosis, in which we can at least demonstrate the presence of what seems to be a specific bacillus. A few years ago consumption, as it is still popularly understood, was a distinct disease; tubercular peritonitis and meningitis were distinct diseases; as also were what we were taught to speak of as "white swelling" and "struma."

Now, a missing link has been discovered in the tubercle bacillus, which seems to unite these various conditions, and we group them together under the term tuberculosis. It must not be forgotten, however, that even here we have at best only demonstrated the presence of one factor which is common to these conditions; the other, and probably the far more important of the two—namely, the nature of the soil—still remains an unknown quantity.

Taking tuberculosis, however, as an example, is rheumatoid arthritis a disease even in the sense that it possesses a constant microbe? In the wider acceptance of the term, with those chronic and osteo-arthritic cases, which some include, and some exclude, the answer may at once be given in the negative, for the discoverers of micro-organisms say that these are not found in such cases.

How is it in its more typical and acuter form?

*Schuller* it was, I think, who first demonstrated a micro-organism in rheumatoid arthritis. It was a short bacillus, with polar enlargements, staining readily by ordinary methods, and sometimes accompanied by micrococci and the gonococcus.

*Schneider's* organism was a small micrococcus. In his case the centre of infection was, he believed, an inflamed gall-bladder.

*Raymond* describes a short thin diplo-bacillus, growing only in synovial fluid.

*Drs. Bannatyne* and *Wohlmann* found a small bacillus which grows best in peptone meat broth, and stains darkly at both ends, but with some difficulty. The culture, by the way, in this case has the appearance of "gold dust," which confirms the opinion I have long held that microbes are a good investment.

We have thus four distinct micro-organisms from which to choose, differing from each other, not only in the outward form, but also in their dietary, and choice of colours. We are told that each in turn is the true germ of rheumatoid arthritis, and the natural conclusion is that they, none of them, are its specific cause.

The evidence so far, then, in this direction, does not tend to establish rheumatoid arthritis as a distinct disease. If we turn back to its clinical features, and enquire into the past history of the cases, we are equally met with an absence of uniformity and a state of chaos. We find rheumatoid arthritis running into, and overlapping, other diseases in a manner which is perfectly bewildering. In what is known as gonorrhœal rheumatism, and as a result of gout, and of abscess in the lung or in the middle ear, we see typical cases of rheumatoid arthritis.

Dr. Cave quoted, at the Cheltenham meeting, a case where all the symptoms of the disease followed in the course of a severe ulceration of the rectum. Then in tetany we have a disease which is sometimes indistinguishable from rheumatoid, where the periarticular swellings and the puffy enlargement, especially on the dorsum of the hands, suggest a close connection between these two conditions.

In Raynaud's disease, too, as Dr. Llewellyn Jones has lately pointed out, in Graves' disease, to which attention was called by Dr. Spender, and in simple anæmia, the similarity equally exists. The evidence to be drawn from all this points strongly, I think, to two things: first, that rheumatoid arthritis is not a distinct disease, due to any one specific organism; and secondly, that it is a condition of ill health resulting from various infective poisons. There is one disease, the influence of which in the causation of rheumatoid arthritis it has been rather the fashion lately to decry. I mean rheumatism. This is a disease which probably possesses a special micro-organism. Ten years ago Dr. Buzzard suggested that this would prove to be the fact, and that it would be shown that this organism acted through the nervous centres. Recently Drs. Poynton and Paine have described a diplococcus which they regard as the cause of rheumatism: whether theirs is the true one or not, I think we may assume that one exists. Rheumatism in its more regular forms varies much in its symptoms, and in the degree and extent of its attacks.

I have seen undoubted cases of acute rheumatic inflammation of the nerves, and of the meninges of the brain and spinal spiral cord; and I think the view may fairly be maintained that the acuter manifestations of the rheumatic poison have also their counterpart in the more chronic forms, such as sciatica, lumbago, and chronic joint affection; and I would extend its influence still further, so as to include many cases of the so-called rheumatoid arthritis.

I was glad to notice that at the Cheltenham meeting Dr. Garrod apparently agreed with the view I took in a treatise I had published about six months previously, namely, that rheumatoid arthritis was probably an irregular manifestation of the rheumatic state. I feel more strongly than ever that this view is right, only with this important modification, that, instead of saying this of rheumatoid arthritis, I should now say it only of a large number of those cases which we have in the past grouped together as a distinct disease, and called by that name.

I regard these cases as due to the specific organism of rheumatism finding itself in soil of a special kind, and developing, not the true disease, but the bastard form called rheumatoid arthritis.

The association, clinically and in the previous history of rheumatism with rheumatoid arthritis is too intimate to be dismissed merely as a coincidence. When we look at the nervous side of rheumatism, and see its connection with another nervous disease—namely, chorea,—and remember the marked nervous phenomena of rheumatoid arthritis, I think that it is fair to assume that, in many cases at least, the results are brought about by the specific micro-organism of rheumatism acting through the nervous system, and producing what may be called a rheumatic nervous illness.

Is this theory, of different results brought about by a common microbe in different soils, too fanciful? I think not. Does it not occur in tuberculosis, which has been mentioned? Are not the tubercular joint and the pulmonary phthisis but the result of the tubercle bacillus acting differently according to the individual susceptibility, or, in other words, according to the difference of the soil?

The variation in the behaviour of cultured microbes is very suggestive of what they may do in their wild condition. If differences can be observed with the artificial feeding in the laboratory, how much more may they not occur in the laboratory of the human body?

Is it too great a stretch of the imagination to suppose that apparently dissimilar bacilli may be descendants of a common ancestor who have developed differently in their different surroundings, or even that micrococcus and bacillus are at times but varying phases in the life-history of the same organism? We have an analogy in nature which at least suggests this possibility. Who, seeing ten feet of the solitary tapeworm on the table, and, side by side with it, its tiny cysticercus, would guess, unless he knew, that they were but stages in the life-history of the same individual?

The case of the caterpillar, the chrysalis, and the butterfly is a prettier instance, perhaps, of individual continuity through many forms. If this polymorphic theory as applied to micro-organism is correct, it may help to explain some of the different germ-forms that have been found by various observers in rheumatoid arthritis. But the very idea of its possibility should make us pause, and be less inclined to dogmatise as to the true position of microbes in the causation of disease.

Take typhoid fever, for instance. Is it necessary for the bacillus typhosus to find entrance to the body from without for this to be produced? Or cannot the bacillus coli develop, when the conditions are favourable, into the more virulent typhoid bacillus within the body?

Sporadic cases of this disease, such as the single one that occurred in Bath last year—which apparently came from nowhere, and led to nothing more,—seem to suggest that this is at least a possibility. We can educate the bacillus coli by artificial means till it does not produce indol or gas in its growth, and until it no longer is capable of coagulating milk. In this condition it resembles, in these respects at least, the typhoid bacillus. Moreover, this latter microbe has been taught to produce gas and indol, and to coagulate milk like the bacillus coli. These things being so, is there anything extraordinary in supposing—even in the absence of present evidence—that, within the body in its natural state, the bacillus coli may become the cause of typhoid fever?

There was one bacillus typhosus a little time ago; now there are many strains of this microbe which each respond in turn to their respective Widal's test, and as a means of diagnosis the negative evidence of this test has now ceased practically to be of any value. The influence of the soil in modifying the functions of micro-organism is to be noticed, too, in the bacillus prodigiosus. When this organism has grown old in the temperature of the room it loses its pigment-producing powers, but these can be restored again by growing it on a potato.

Shortly to sum up, then, the evidence to be found in the previous history, the exciting causes, the clinical features, and the bacteriology of rheumatoid arthritis all point away from this being any longer recognised as a distinct disease. I look upon it as a condition due, not to any one specific germ, but to various different kinds of germs, which link it to rheumatism, to rheumatic arthritis, to gout, and to other diseases, some of which I have mentioned; and I suggest that these organic poisons, either by themselves or through their toxins, produce their results by acting through the nervous centres in an individual whose vitality is lowered and whose joints have a tendency to disease.

